# Chamber volume development, metabolic rates, and selective extinction in cephalopods

**DOI:** 10.1038/s41598-020-59748-z

**Published:** 2020-02-19

**Authors:** Amane Tajika, Neil H. Landman, René Hoffmann, Robert Lemanis, Naoki Morimoto, Christina Ifrim, Christian Klug

**Affiliations:** 1 Division of Paleontology (Invertebrates), American Museum of Natural History, Central Part West 79th Street, New York, NY 10024 USA; 2 Institut für Geologie, Mineralogie und Geophysik, Ruhr-Universität Bochum, Bochum, 44801 Germany; 3 BCUBE – Center for Molecular Bioengineering, Technische Universität Dresden, Dresden, 01307 Germany; 4 Laboratory of Physical Anthropology, Graduate School of Science, Kyoto University, Kitashirakawa Oiwake-cho, Sakyo-ku, 606-8502 Kyoto Japan; 5 Institut für Geowissenschaften, Ruprecht-Karls-Universität Heidelberg, Im Neuenheimer Feld 234/Raum 217, D-69120 Heidelberg, Germany; 6 Paläontologisches Institut und Museum, Universität Zürich, Karl-Schmid-Strasse 4, Zürich, 8006 Switzerland

**Keywords:** Palaeontology, Palaeoecology

## Abstract

Reconstructing the physiology of extinct organisms is key to understanding mechanisms of selective extinction during biotic crises. Soft tissues of extinct organisms are rarely preserved and, therefore, a proxy for physiological aspects is needed. Here, we examine whether cephalopod conchs yield information about their physiology by assessing how the formation of chambers respond to external stimuli such as environmental changes. We measured chamber volume through ontogeny to detect differences in the pattern of chamber volume development in nautilids, coleoids, and ammonoids. Results reveal that the differences between ontogenetic trajectories of these cephalopods involve the presence or absence of abrupt decreases of chamber volume. Accepting the link between metabolic rate and growth, we assume that this difference is rooted in metabolic rates that differ between cephalopod clades. High metabolic rates combined with small hatching size in ammonoids as opposed to lower metabolic rates and much larger hatchlings in most nautilids may explain the selective extinction of ammonoids as a consequence of low food availability at the end of the Cretaceous.

## Introduction

The Ammonoidea is a group of ectocochleate cephalopods that were extant for more than 350 million years, during which time they played an essential ecological role in the world’s oceans as a result of their high abundance, wide distribution, and great diversity. Although they survived several of the most severe mass extinction events in the course of their evolution^1–3^, they perished at the end of the Cretaceous^4,5^. Despite extensive discussion on the selectivity of the K/Pg extinction, the actual mechanisms that led ammonoids to extinction and allowed nautilids to survive, have not yet been fully revealed, although both intrinsic (e.g., smaller embryonic sizes, larger geographical range, and microphagous feeding^4,6,7^) and extrinsic factors (e.g., surface ocean acidification and global cooling^8,9^) have been proposed. Details about intrinsic (anatomical and physiological) aspects such as the muscular system and metabolic rates are difficult to assess in extinct organisms because the soft tissue is rarely fossilized^10^. Thus, we need proxies for biological and physiological aspects to fully reveal the actual kill mechanism of ammonoids at the K/Pg boundary. In fact, such biological and physiological traits are apparently strongly linked to macroecology and macroevolution of organisms. For instance, Strotz *et al*.^11^ discovered a significant difference between basal metabolic rates of extinct and extant taxa. Additionally, Payne *et al*.^12^ demonstrated a new perspective on the evolution of bivalves and brachiopods by calculating their metabolic rates. Reconstructing the biological and physiological traits of extinct ammonoids may, therefore, be a key to understanding selective extinction^13^.

Most mollusk conchs contain a wealth of information about their development because the entire life history is recorded within the shell. In ectocochleate cephalopods (ammonoids and nautiloids), the conchs, which comprise the gas-filled phragmocone and the soft-tissue-bearing body chamber, have been studied with a focus on the external morphological characters such as ornamentation and coiling^14,15^. The internal structure of the conchs, however, has been studied much less frequently, largely due to technical difficulties of analyzing the often recrystallized and more or less sediment-filled conchs. Among other parameters, septal spacing of ammonoids, nautilids, and belemnites is of great interest because septa and chambers are constructed by the soft tissues of the animal, and may, therefore, provide information about key aspects of life history such as hatching, growth changes, and mode of life^16–19^. In addition to the conventional 2D-analyses of septal spacing through ontogeny (i.e., measuring septal rotational angles^16,20–22^), recent destructive and non-destructive methods to three-dimensionally reconstruct chamber volume have been developed^23–26^. Obtaining 2D-data is advantageous because of the simple preparation of fossils (grinding and polishing), requiring minimal lab time and post-processing; however, the changes of septal angle through ontogeny are sometimes very subtle, and thus this method may mask some important details. By contrast, although the 3D-method requires relatively complex technical set-ups to produce image stacks (e.g., high-energy beams for fossils in X-ray computed tomography, which is non-destructive or grinding tomography for low-contrast materials, which is destructive) and post-processing of image stacks is considerably time-consuming, the resulting volumetric data through ontogeny provide valuable information that might not be obtained from 2D-data. By plotting such 3D-measurements, we obtained curves that approximately conform to exponential functions, emphasizing subtle ontogenetic changes in septal spacing^23–26^ (see Naglik *et al*.^24^ and Hoffmann *et al*.^27^ for comparisons of 2D- and 3D-data). These studies reveal various patterns of ontogenetic change in septal spacing in several cephalopod taxa. However, the factors that determine these patterns and, particularly, the differences are hardly known. Some authors have suggested possible links between ecological changes (such as habitat changes) and abrupt changes in septal spacing, although such studies are still limited^16,28,29^. Determining the factors that control the pattern of septal spacing and growth of chamber volume can be of great relevance because they may be widely applicable to better understand environmental, ecological, and biological aspects of these organisms.

In this study, we depict volumetric growth trajectories of phragmocone chambers in various cephalopod taxa. We address the question of whether the development of chamber volume in cephalopod phragmocones conveys information about their physiology. We examine some specimens that bear various degrees of pathology to further discuss what controls the pattern of chamber volume development. We aim to answer the following questions:1) What are the typical patterns of chamber volume development in various cephalopod groups and how do they differ? 2) How do pathologies affect chamber volume development? 3) What are the factors that alter patterns of chamber volume development between different cephalopod groups? 4) What do these factors tell us about the ecology and extinction selectivity of cephalopods?

## Methods

**Figure 1:**
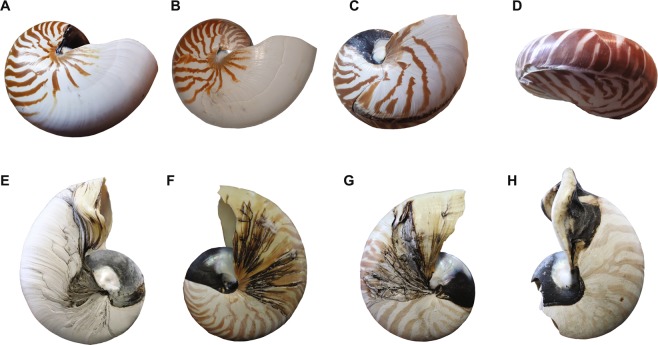
Specimens of *Nautilus pompilius* with differing degrees of pathology examined in this study. (**A**) RUB-Pal 11248 (conch diameter = 180 mm) with a moderate pathology. (**B**) RUB-Pal 11267 (conch diameter = 187 mm) with a moderate pathology. (**C**) RUB-Pal 11270 (conch diameter = 148 mm) with a moderate pathology. (**D**) RUB-Pal 11268 (conch diameter = 83 mm) with a fatal pathology (pathology that led to premature death after phase of ill health). (**E–H**) AMNH FI 63303–63306 (conch diameters = 120, 110, 113, 132 mm, respectively), aquarium-reared specimens with a fatal pathology (pathology that led to premature death after a phase of ill health). RUB-Pal = Ruhr-University Bochum Palaeontology. AMNH FI = American Museum of Natural History Fossil Invertebrates.

We studied 24 cephalopod conchs: 15 conchs of modern nautilids (2 with no pathology, 13 with pathology including 7 aquarium-reared individuals; Fig. 1), 3 specimens of the Cretaceous nautilid *Eutrephoceras nebrascensis* (1 specimens from the upper Campanian *Baculites compressus* Zone in Montana; 2 specimens from the *B. compressus* Zone in South Dakota), 4 specimens of Cretaceous ammonites (3 specimens of *Tetragonites* sp. and 1 specimen of *Gaudryceras* sp. from the Campanian of Hokkaido, Japan), 2 specimen of the modern coleoid *Spirula spirula* (see Table 1 for more detailed information). The specimens of the fossil nautilids and ammonites did not display any deformities or pathologies on the conchs with the exception of *Tetragonites* (NMA00803), where the shell dissolved during diagenesis^30^. In this study, we divide the degrees of pathology into ‘moderate’ and ‘fatal’. The former indicates that the animals still continued to grow and reached maturity in spite of the pathology. By contrast, the latter are those, which (most likely) continued to live for some time after the incident, but died before reaching maturity.

**Table 1:** Details of the examined specimens.

	Species	Specimen number	Age	Locality	Pathology	Conch diameter (mm)	3D reconstruction method	Voxel size (mm)
nautilid	*Allonautilus scrobiculatus*	RUB-Pal 11247	Modern	unknown	no	150?	computed tomography	0.060*0.060*0.060
	*Nautilus pompilius*	PIM 7825			no	166		0.091*0.091*0.091
		RUB-Pal 11248			moderate	180		0.089*0.089*0.089
		RUB-Pal 11266			moderate	174		0.085*0.085*0.085
		RUB-Pal 11267			moderate	187		0.099*0.099*0.099
		RUB-Pal 11268			fatal	83		0.040*0.040*0.040*
		RUB-Pal 11270			moderate	148		0.079*0.079*0.079*
		RUB-Pal 11271			moderate	168		0.083*0.083*0.083
		Zoo Arnhem, coll. AWI Bremerhaven; coll. no. Nehrke 01		unknown (aquarium)	fatal	136		0.087*0.087*0.087
		Bochum Tierpark 01			fatal	148		0.087*0.087*0.087
		Bochum Tierpark 02			fatal	164		0.088*0.088*0.088
		AMNH FI 63303			fatal	120		0.053*0.053*0.053
		AMNH FI 63304			fatal	110		0.053*0.053*0.053
		AMNH FI 63305			fatal	113		0.053*0.053*0.053
		AMNH FI 63306			fatal	132		0.060*0.060*0.060
	*Eutrephoceras nebrascensis*	AMNH FI 102486	Campanian	Pierre Shale, ?Montana	no	12		0.010*0.010*0.010
		SD 002		Pierre Shale, South Dakota	no	37	grinding tomography	0.010*0.010*0.160
		SD 003			no	31	grinding tomography	0.010*0.010*0.160
ammonoid	*Tetragonites* sp.	NMA00803	Campanian	Haborogawa Fm., Hokkaido	unknown	36	computed tomography	0.020*0.020*0.020
		HKD TG 001			no	20	grinding tomography	0.010*0.010*0.070
		HKD TG 002			no	25	grinding tomography	0.010*0.010*0.070
	*Gaudryceras* sp.	HKD GC			no	28	grinding tomography	0.010*0.010*0.070
coleoid	*Spirula spirula*	PIMUZ 017853 PIMUZ 37573	modern	unknown	no	22 17	computed tomography	0.018*0.018*0.018 0.033*0.033*0.033

RUB-Pal = Ruhr-University Bochum Palaeontology. AMNH FI = American Museum of Natural History Fossil Invertebrates. PIMUZ = Palaeontological Institute and Museum, University of Zurich. NMA = Nakagawa Museum of Natural History.

In order to extract chamber volume through ontogeny, we three-dimensionally reconstructed the conchs. To this end, computed tomography was applied to the modern nautilid specimens and one specimen each of *Tetragonites* sp. and *Eutrephoceras* sp., while grinding tomography was performed on the other fossils (for details of the procedure for grinding tomography, see Naglik *et al*. and Tajika *et al*.^24,31^). The images obtained with grinding tomography were sharpened and the contrast was enhanced. The image stacks obtained were segmented in Avizo 8.1 (Volume Graphics) to export the shell surface, which was then inverted in Meshlab (ISTI - CNR research center) to extract the phragmocone. The phragmocone was decomposed into individual chambers and the respective chamber volumes were measured using MATLAB (MathWorks). The chamber volumes measured were plotted against chamber number through ontogeny with chamber 1 as the first formed chamber after the protoconch. Because the first several chambers were not clearly visible due to insufficient contrast of our image stacks, the exact chamber number in ammonoids was unknown.

To examine the pattern of chamber volume development within a specimen, we calculated the chamber volume development rate (=volume of a chamber/volume of the proceeding chamber). If chamber volume increases, as expected from the allometric or isometric growth of cephalopods, the chamber volume development rate is higher than 1.0. The chamber volume development rates were also plotted through ontogeny.

## Results

### General trend of chamber volume development

**Figure 2:**
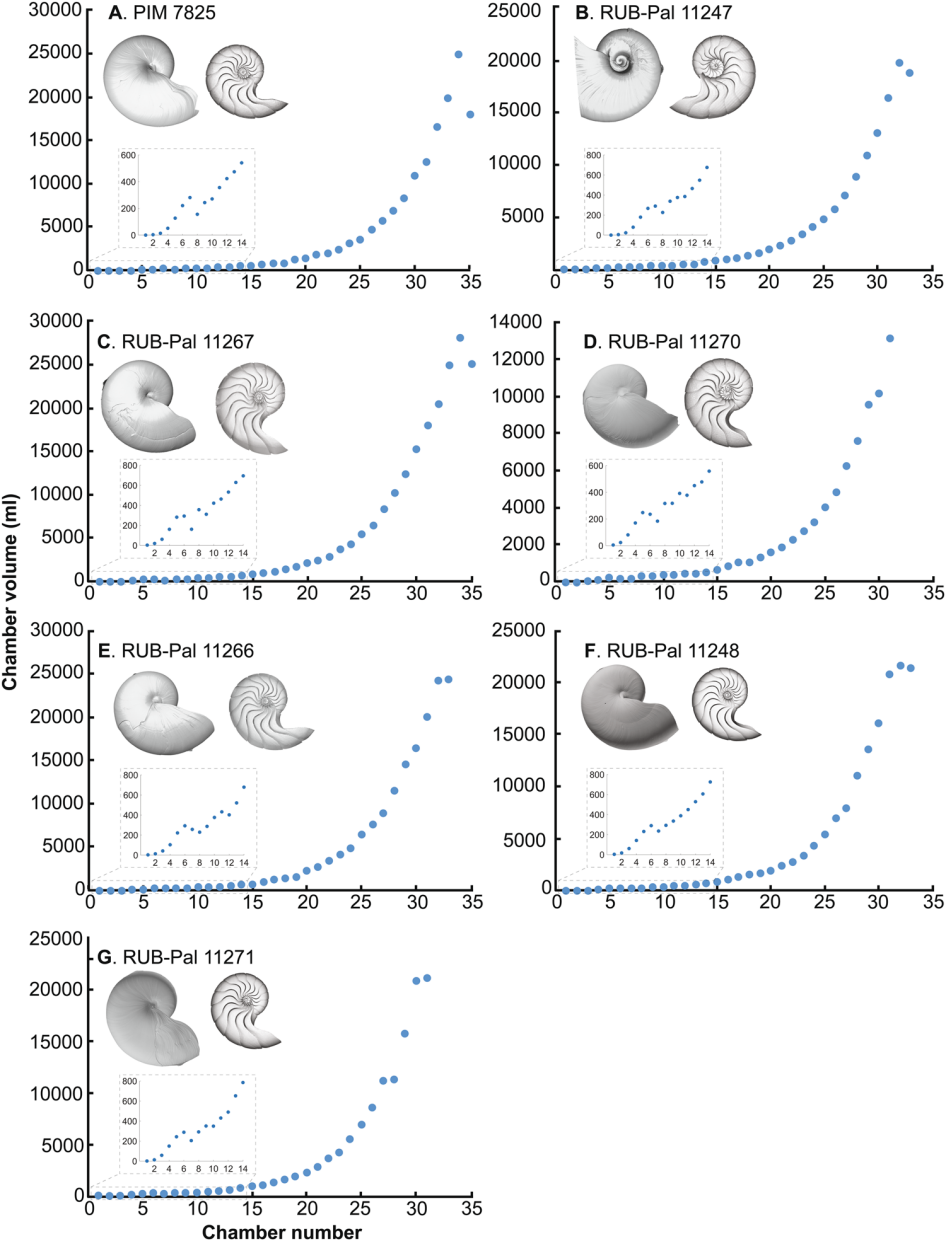
Volumtric growth trajectories of modern nautilids with no and moderate pathology. (**A**), (**C)**-(**O)**, *Nautilus pompilius*. (**B**) *Allonautilus scrobiculatus*. (**A**) PIM 7825, no pathology. (**B**) RUB-Pal 11247, no pathology. (**C**) RUB-Pal 11267, moderate pathology. (**D**) RUB-Pal 11270, moderate pathology. (**E**) RUB-Pal 11266, moderate pathology. (**F**) RUB-Pal 11248, moderate pathology. (**G**) RUB-Pal 11271, moderate pathology.

**Figure 3:**
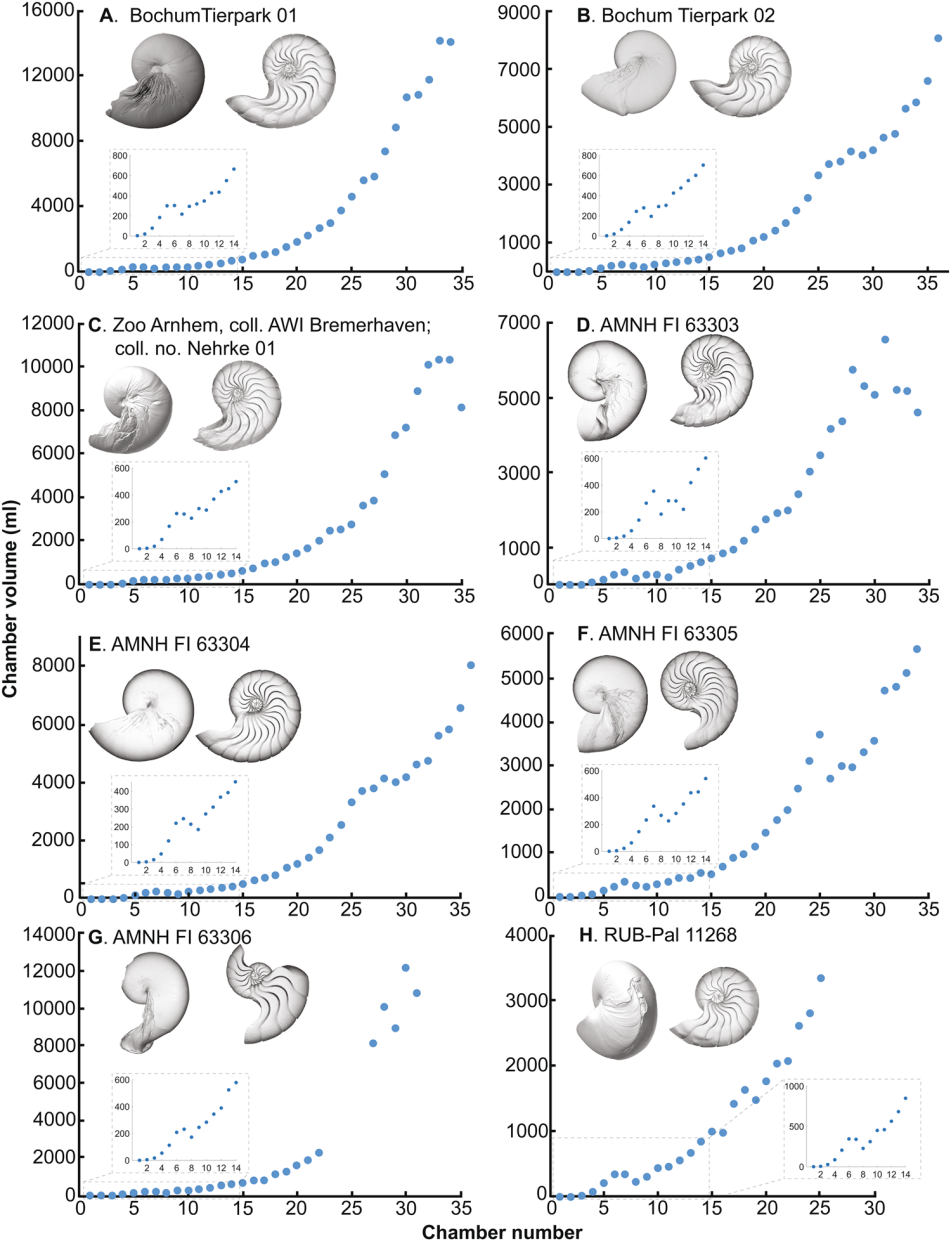
Volumtric growth trajectories of modern nautilids (*Nautilus pompilius*) with fatal pathology (pathology that led to premature death). (**A**) Bochum Tierpark 01. (**B**) Bochum Tierpark 02. (**C**) Zoo Arnhem, coll. AWI Bremerhaven; coll. no. Nehrke 01. (**D**) AMNH FI 63303. (**E**) AMNH FI 63304. (**F**) AMNH FI 63305. (**G**) AMNH FI 63306. (**H**) RUB-Pal 11268.

**Figure 4:**
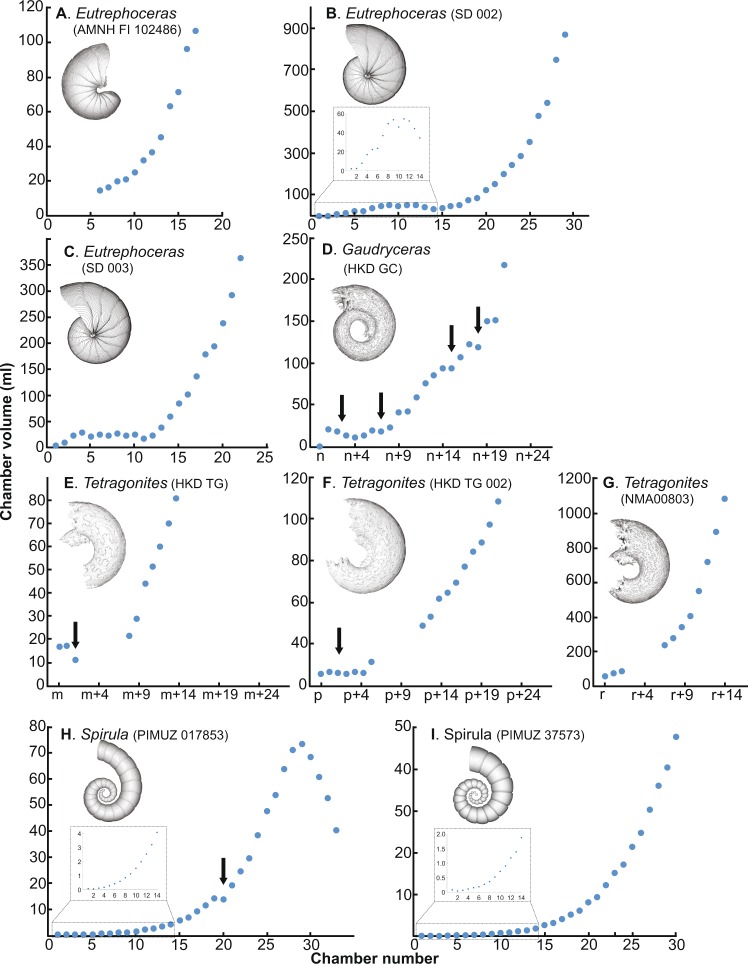
Volumetric growth trajectories in Cretaceous nautilids (**A–C**), ammonites (**D–G**), and modern coleoid **(H**,**I**). (**A**) *Eutrephoceras nebrascensis* (AMNH FI 102486), (**B**) *E. nebrascensis* (SD 002). (**C**) *E. nebrascensis* (SD 003). (**D**) *Gaudryceras* sp. (HKD GC). (**E**) *Tetragonites* sp. (HKD TC 001), (**F**) *Tetragonites* sp. (HKD TC 002). (**G**) *Tetragonites* sp. (NMA00803). (**H**) *Spirula spirula* (PIMUZ 017853). (**I**) *S. spirula* (PIMUZ 37573). PIMUZ = Palaeontological Institute and Museum, University of Zurich. NMA = Nakagawa Museum of Natural History. Specimens (**B–F**) are no longer available due to destructive sampling.

**Figure 5:**
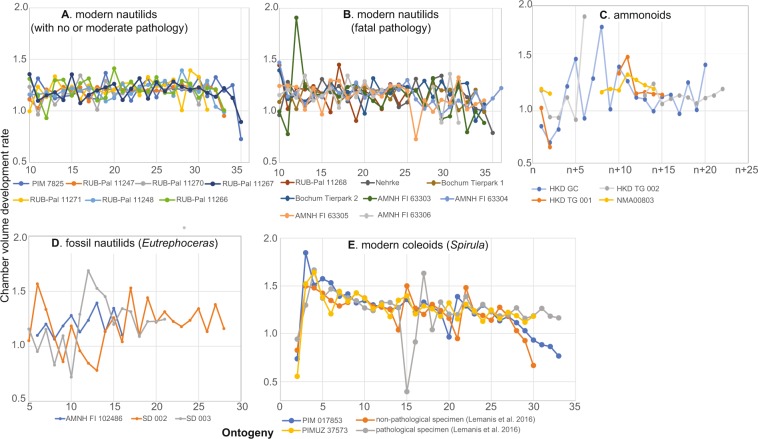
Chamber volume development rates through ontogeny in nautilids, ammonoids, and coleoids. (**A**) Modern nautilids (*Allonautilus scrobiculatus* and *Nautilus pompilius* with no or moderate pathology). (**B**) Modern nautilids (*Nautilus pompilius* with fatal pathology: pathology that led to premature death). (**C**) Ammonoids (*Tetragonites* and *Gaudryceras*). Note that chamber volume in each specimen was not measured in the same ontogenetic stage. (**D**) Fossil nautilids (*Eutrephoceras*) (**E**) modern coleoid (*Spirula spirula*; data of two specimens (with and without pathology) from Lemanis *et al*. (2016).

Three-dimensionally reconstructed conchs and phragmocone chambers as well as phragmocone chamber volumes are shown in Figs. 2–4 (Supplementary Table 1). The chamber volume development rates in all the examined specimens are shown in Fig. 5 (Supplementary Table 1). In modern nautilids with and without pathology, chamber volumes show an increasing trend although individuals with a pathology leading to premature death often exhibit fluctuations. Such fluctuations appear to account for the difference in total phragmocone volume between individuals with no and moderate pathology and those with fatal pathologies (Figs. 2, 3, and Supplementary Table). Chamber volume in the embryonic and post-hatching stages (up to chamber 13) also fluctuates in nautilids. Although the earliest ontogenetic stages are missing, chamber volume in the ammonoids and the modern coleoid *Spirula* shows an increasing trend. The volumetric growth trajectories of the ammonoids and coleoid show abrupt decreases of chamber volume through ontogeny (Figs. 4D–F,H, 5C,E). In *Spirula*, chamber volume also decreases during the latest ontogeny (chambers 30–34). This decrease toward the end of ontogeny is probably related to the attainment of maturity^22^.

### Pattern of chamber volume development in modern nautilids

Fluctuations in chamber volume development appear to occur only in individuals with pathologies that led to premature death (Fig. 3) with the exception of embryonic, post-hatching, and mature stages, in which septal crowding in modern *Nautilus* is known^23,25,32,33^. The chamber volume development rate in non-pathological and moderately pathological individuals ranges mostly between 1.0–1.5 (Fig. 5). By contrast, the aquarium-reared individuals with pathologies that led to premature death often show abrupt decreases in volumetric growth trajectories (chamber volume development rates <1.0). These results are consistent with an experimental study on *Nautilus* by Keupp and Riedel^29^, in which they discovered that septal crowding occurs in aquarium-reared individuals as a reaction to adverse conditions rather than to the attainment of maturity. This illustrates the general pattern of chamber volume development in modern nautilids: only a pathology that leads to early death strongly affects the formation of chambers (Fig. 3) while slight pathologies (injuries, illnesses, adverse conditions) do not produce disharmonic chamber growth trajectories (Fig. 2). Considering that seven out of eight fatally pathological individuals are aquarium-reared, in which the ecological conditions significantly differ from that of nature (e.g., shallow water depth implying low hydrostatic pressure), it appears that the chamber volume development rate of modern nautilids in nature consistently increases even in cases where specimens display moderate pathologies. The consistent chamber volume development of *Nautilus pompilius* in nature was already reported by Tajika *et al*.^25^.

### Pattern of chamber volume development in fossil nautilids

As in their modern relatives, the specimens of the Cretaceous nautilid *Eutrephoceras* show a similarly fluctuating pattern in early ontogeny (up to chamber 13; Figs. 4B,C, 5D). The changes of septal spacing at the embryonic stages in *Eutrephoceras*, which coincide with hatching, are well documented^17,34^. Later in ontogeny, the chamber volume increases relatively constantly at a rate between 1.0–1.5. This indicates that the Cretaceous nautilid *Eutrephoceras* and modern nautilids share a similar pattern of chamber volume development, in which both groups construct chambers with a consistently positive chamber volume development rate under normal natural conditions (i.e., when not excessively stressed). To date, no data on septal spacing in pathological fossil nautilids are available.

### Pattern of chamber volume development in fossil ammonoids and coleoids

Due to insufficient contrast, resolution and preservation, the chambers formed during the earliest part of ontogeny could not be segmented with enough accuracy. Thus, our data on ammonoid chamber volume development were taken only from juvenile post-hatching growth stages (for the size of each specimen, see Table 1). Nevertheless, a difference in the pattern of chamber volume development appears to exist between ammonoids and nautilids. Although the studied ammonoid specimens HKD GC, HKD TG, and HKD TG 002 do not display any trace of pathology on the conchs, they show decreases in chamber volume development (distinct in *Gaudryceras* and *Tetragonites* in Fig. 4D,E, respectively; less conspicuous in *Tetragonites* in Fig. 4F). Naturally, these decreases are reflected in negative chamber volume development rates (Fig. 4B). In contrast, NMA00803 (*Tetragonites*) does not show such abrupt decreases during ontogeny (Fig. 4G). Naglik *et al*.^24^ published volumetric growth trajectories of two Devonian ammonoids (*Diallagites* and *Fidelites)* and one Carboniferous ammonoid (*Goniatites*). Although not discussed in that article, the graphs show high fluctuations during ontogeny. We calculated the chamber volume development rates for the ammonoid data of Naglik *et al*.^24^, which revealed that chamber volume decreases repeatedly at different ontogenetic stages (Supplementary Table) in the absence of distinct pathologies. Tajika *et al*.^25^ published volumetric data based on grinding tomography of two specimens of the Jurassic ammonoid *Normannites*. In their study, one of the specimens bears a *syn vivo* epizoan but does not show abrupt changes in chamber volume development, whereas an abrupt reduction of chamber volume occurred in the other specimen with no visible pathology or epizoan. Lemanis *et al*.^23^ studied one Carboniferous (*Arnsbegites*) and two Jurassic ammonoids (*Cadoceras* and *Amauroceras*), in which abrupt reductions of chamber volume also occurred. Our data and those of the previous studies confirm that abrupt changes of chamber volume (i.e., negative chamber volume development rates prior to maturity) are common among all ammonoids from the Devonian to the Cretaecous with few exceptions^25^ (Fig. 4G).

The modern coleoid *Spirula spirula* appears to have a pattern similar to that of ammonoids. In the ontogenetic trajectories of chamber volumes (Fig. 4H), an abrupt decrease occurs in the middle of ontogeny, which naturally corresponds to a negative chamber volume development rate in Fig. 5E. Lemanis *et al*.^23^ illustrated the volumetric trajectories in pathological and non-pathological specimens of *Spirula spirula*. The chamber volume development rate shows a negative value in both specimens, although the pathological specimen shows a higher rate (Fig. 5E). As in ammonoids, *Spirula spirula* displays abrupt changes in volumetric growth trajectories regardless of the presence or absence of pathologies with some exceptions (Fig. 4I).

## Discussion

We discovered the following patterns of chamber volume development:

- In modern and fossil nautilids, chamber volume usually increases constantly during ontogeny without abrupt drops under normal and natural environmental conditions. Abrupt drops in chamber volume occur only under extreme conditions (e.g., when individuals are reared in an aquarium); most of the examined nautilids collected from the wild do not show such irregular fluctuations in chamber volume.
- In ammonoids and the modern coleoid *Spirula*, chamber volume development rate is roughly constant but it sometimes shows abrupt drops under natural ecological conditions during ontogeny. In *Spirula*, pathological individuals also show a higher rate of abrupt drops of chamber volume.

Although the mode of life (locomotion, migration, physiology) and ecological conditions (temperature, hydrostatic pressure, food availability, and chemical composition of the water) of these organisms cannot be fully reconstructed, we discuss possible factors that could explain these differences in the pattern of chamber volume development.

Change in mode of life: Changes in septal spacing in the earliest ontogeny of nautilids and *Spirula* correspond to hatching, which is also reflected in changes in carbon and oxygen isotopes of the conch^17,22,35,36^. In addition, Arai and Wani^16^ suggested that changes of septal spacing (two-dimensional rotational angles) in Late Cretaceous ammonoids from Japan, which occur at a shell diameter of less than 5 mm, may be linked to the change from a planktic to a more active nektic lifestyle. However, the abrupt decreases of chamber volume in our ammonoid data occur at much larger conch diameters (>10 mm), which suggests that these decreases cannot be explained only by a change of lifestyle.

Change in habitat (environmental conditions): Kraft *et al*.^20^ examined septal spacing of Carboniferous ammonoids from Algeria. They also documented abrupt changes in septal spacing (two-dimensional rotational angles). They concluded that these cases of septal crowding did not indicate maturity (because septal spacing normalized afterward) and was presumably caused by adverse ecological conditions such as low oxygen conditions, poor food availability or toxic chemical composition of the sea water. Some studies discovered positive correlations between ecological factors and lamellar (i.e., septal) spacing in the modern cuttlefish *Sepia officinalis*. For instance, Wiedmann and Boletzky^28^ documented that lamellar spacing in *S. officinalis*, which strongly correlates with growth rate, decreases in phases of very poor food availability. Also, some studies found that lamellar spacing in *S. officinalis* is controlled by temperature^37,38^. Gutowska *et al*.^39^ carried out an experimental study in which they found that cuttlebones of CO_2_- incubated *Sepia officinalis* individuals produce narrower lamellar spacing.

The abrupt changes of chamber volume, which occur in ammonoids and *Spirula*, may also be explained by disadvantageous environmental factors. Nevertheless, such adverse environmental conditions should also affect nautilids. Considering water temperature, it is known that modern *Nautilus* migrates diurnally from deep to shallow water environments between 100–700 m^40^, through which they traverse a temperature gradient. Additionally, as far as ecological conditions are concerned, Cretaceous *Eutrephoceras* presumably inhabited a shallow water environment ~70 m deep^41^, which was probably a higher-energy setting compared to the deep water habitat in which *Spirula* lives around 400–1000 m^36^. The habitat of the Cretaceous ammonoids examined is considered to be an outer shelf setting (near the continental slope), which was supposedly deeper than the habitat of *Eutrephoceras*. Thus, it is assumed that *Eutrephoceras* may have faced more environmental perturbations than the ammonoids and possibly *Spirula*. If temperature and/or environmental perturbations equally affect the septal spacing in nautilids, ammonoids, and coleoids, abrupt decreases of chamber volume should be visible in nautilids. Since our data on nautilids that lived under natural conditions show no fluctuations in volumetric growth trajectories, we suspect that environmental changes alone cannot explain the differences in the patterns of chamber volume development between nautilids, ammonoids, and coleoids.

Differences in metabolic rates (i.e., the minimum energy required to sustain life): The metabolic rate is known to be the rate of energy uptake, transformation, and allocation^42^. Acquisition and processing of energy are essential parts of animal physiology, which generate behavior (e.g., muscle contraction) and new biomass (e.g., growth and egg production^43^). Furthermore, most organisms display phenotypic plasticity in the expression of metabolism as a reflection of environmental differences^42^. For instance, Zeng *et al*.^44^ discovered that individuals with a high metabolic rate within a fish population experienced more mass loss during food deprivation.

As far as metabolic rates of cephalopods are concerned, the ‘live fast, die young’ strategy of many coleoids is well-known and some squids, octopuses, and cuttlefish are metabolically very active although variation in metabolic rate is also significant^45–47^. Modern *Nautilus* is known to live quite long (up to approximately ~20 years^48^), to have a low energy consumption, and to be able to maintain a low metabolic rate^49–51^. Although metabolic rates of *Spirula spirula* are not known, stable carbon isotopes of *Spirula* and *Nautilus* shells, which are considered to reflect metabolic rates in mollusks, suggest a lower δ^13^C value in *S. spirula* than in *Nautilus*^52–54^, and thus a higher metabolic rate. These facts and our new data suggest a possible link between metabolic rates and patterns of chamber volume development in cephalopods: abrupt drops in chamber volume occur in the modern coleoid *Spirula* with a higher metabolic rate, but not in nautilids with a lower metabolic rate.

Presumably, the high energy requirements of coleoids make them more susceptible to adverse ecological conditions, which, in turn, may affect the phenotype (chamber construction) since energy acquisition and processing are essential parts of growth and biomass production. This hypothesis is concordant with the abovementioned experiment by Wiedmann and Bolezky^28^. If this holds true for all phragmocone-bearing cephalopods, the fact that our ammonoid data show abrupt decreases in volumetric growth trajectories as in *Spirula* may suggest a metabolic rate in ammonoids higher than that in nautilids. Such a relatively high metabolic rate of ammonoids coincides with their supposedly relatively good locomotory capabilities and closer phylogenetic relationships to coleoids^55–58^. The actual environmental factors, which induced these abrupt changes, are difficult to detect.

At the end of the Cretaceous, ammonoids went extinct while nautilids survived^4^. After the asteroid impact and the Deccan trap-eruptions^59^, acidification of sea water occurred, which presumably caused a dramatic decrease in the abundance of primary producers and planktic animals^8,60^, thereby drastically cutting the food supply of ammonoids. It is likely that metabolic rate determined the degree to which the respective species could survive food-impoverished times. Assuming that ammonoids possessed a high metabolic rate, they required more energy input per time unit, and, in turn, were particularly susceptible to such adverse conditions. This prevented the soft body from growing and reduced the body chamber volume required for the soft parts, thus lowering shell secretion at the aperture. As a consequence, septal spacing was reduced and smaller chambers were constructed.

Reduced food availability implies a lower energy availability, which particularly affected young individuals, like hatchlings, because of their lower energy reservoirs. In contrast to small ammonoid hatchlings (<2 mm), larger nautilid hatchlings (>10 mm) could maintain their growth with their low metabolism (or by lowering their metabolic rate even more) and survive prolonged phases of low food-availability. Although there are other conceivable factors, which may have contributed to ammonoid extinction^5^, a high metabolic rate of ammonoids, in combination with their very small hatching size, was probably a fatal combination during times of low primary production. By contrast, nautilid hatchlings had larger reserves because they are an order of magnitude larger in diameter and accordingly have a body mass three orders of magnitude larger^7,60,61^. However, some coleoids, which most likely also had a high metabolic rate survived the K/Pg extinction. Although the exact reason for their survival is unclear, their greater range in fecundity, hatching size, locomotory capability, and macrophagous feeding strategy may have protected them from extinction^6,55,62,63^. In any case, belemnites, which also had rather small hatchlings, became extinct, while vampyromorph coleoids (ancestors of modern octopodids), which likely had larger hatchlings, survived^63^. Variation in embryonic size and metabolism in Mesozoic coleoids as well as the exact kill mechanism need further investigation.

## Supplementary information

Supplementary Table 1.
